# Comparative Effectiveness of Antiviral Agents and Monoclonal Antibodies for Early SARS-CoV-2 Therapy in Immunocompromised Patients: A Multicenter Retrospective Cohort Study (March 2021–March 2022)

**DOI:** 10.3390/microorganisms13051076

**Published:** 2025-05-06

**Authors:** Serena Vita, Gaetano Maffongelli, Tommaso Ascoli Bartoli, Domenico Benvenuto, Raffaella Marocco, Silvia Rosati, Valentina Mazzotta, Cosmo Del Borgo, Ilaria Mastrorosa, Patrizia De Marco, Alessandra D’Abramo, Fabrizio Maggi, Andrea Antinori, Miriam Lichtner, Emanuele Nicastri, COVID Group

**Affiliations:** 1 National Institute for Infectious Diseases Lazzaro Spallanzani—IRCCS, 00149 Rome, Italy; serena.vita@inmi.it (S.V.); gaetano.maffongelli@inmi.it (G.M.); tommaso.ascoli@inmi.it (T.A.B.); silvia.rosati@inmi.it (S.R.); valentina.mazzotta@inmi.it (V.M.); ilaria.mastrorosa@inmi.it (I.M.); patrizia.demarco@inmi.it (P.D.M.); alessandra.dabramo@inmi.it (A.D.); fabrizio.maggi@inmi.it (F.M.); andrea.antinori@inmi.it (A.A.); emanuele.nicastri@inmi.it (E.N.); 2 Dipartimento di Sicurezza e Bioetica, Sezione di Malattie Infettive, Università Cattolica del Sacro Cuore, 00168 Rome, Italy; 3 Infectious Diseases Unit, Santa Maria (SM) Goretti Hospital, Sapienza University of Rome, 04100 Latina, Italy; r.marocco@ausl.latina.it (R.M.); c.delborgo@ausl.latina.it (C.D.B.); miriam.lichtner@uniroma1.it (M.L.)

**Keywords:** SARS-CoV-2, COVID-19, immunocompromised patients, antiviral agents, monoclonal antibodies

## Abstract

Immunocompromised (IC) patients continue to be at risk of severe COVID-19 despite vaccination and anti-SARS-CoV-2 therapies. The comparative effectiveness of antiviral agents (AVAs) and monoclonal antibodies (MoAbs) as early treatment of SARS-CoV-2 in IC patients is described in this work. This retrospective multicenter cohort study included IC outpatients diagnosed with SARS-CoV-2 between March 2021 and March 2022 at the National Institute for Infectious Diseases “Lazzaro Spallanzani” and Santa Maria Goretti University Hospital, Italy. Patients received either AVAs or MoAbs based on national guidelines. The primary outcome was time to negative nasopharyngeal swab (NPS). The secondary outcomes were COVID-19-related hospitalization or death by day 30. Among 1472 IC patients (with a median age of 58 years, 45% male), 688 (46%) were treated with MoAbs, and 783 (54%) were treated with AVAs. The patients treated with MoAbs had a higher duration to negative NPS (17 vs. 11 days, *p* < 0.05) and a higher risk of sustained SARS-CoV-2 positivity on day 7 (OR: 3.0, 95% CI: 1.72–5.23, *p* < 0.01) and day 30 (OR: 6.0, 95% CI: 3.7–10.5, *p* < 0.01) than those treated with AVAs. There were no differences in hospitalization or mortality. AVAs were associated with a more rapid viral clearance than MoAbs, suggesting a potential advantage for reducing infectious duration in IC patients. Additional studies are necessary to further optimize the early treatment of COVID-19 in this high-risk population.

## 1. Introduction

Since the beginning of the pandemic of severe acute respiratory syndrome coronavirus 2 (SARS-CoV-2), the severity and mortality of coronavirus disease 2019 (COVID-19) have decreased. This is mainly due to the broad implementation of the SARS-CoV-2 vaccine, the increased availability of antivirals and monoclonal antibodies, SARS-CoV-2 natural infection and reinfection immunity, and attenuated viral pathogenicity [1,2]. Nevertheless, immunocompromised (IC) patients remain at an increased risk of severe COVID-19 and fatal outcomes [3,4]. In the US, IC patients account for 6.6% of the population [5]; in the UK, they account for 3.9% when broadly defined and 0.7% when stringently defined [6]; and they are disproportionately affected by COVID-19 [7]. Clinical management and therapeutic approaches to SARS-CoV-2 infection in IC patients are reported in a rising number of publications, mostly from case series or retrospective cohorts [8,9,10,11,12]. National and international guidelines on COVID-19 clinical management now include a section dedicated to IC patients. Still, most recommendations rely on limited evidence based on limited retrospective case cohorts, few anecdotal cases, and expert opinions only [13,14].

The aim of this longitudinal cohort study is to compare the clinical outcomes of IC outpatients with early SARS-CoV-2 infection treated with antiviral agents (AVAs) or variant-specific monoclonal antibodies (MoAbs).

## 2. Materials and Methods

This longitudinal prospective cohort study involved consecutive patients with a confirmed SARS-CoV-2 infection and a documented history of immune-compromised disease. Patients were enrolled at the Lazzaro Spallanzani National Institute for Infectious Diseases (INMI) in Rome, Italy, or at Santa Maria Goretti University Hospital in Latina, Italy, from 21 March 2021 to 30 March 2022. All COVID-19 patients meeting the inclusion criteria for early antiviral treatment, as defined by the Italian Drug Agency, were enrolled (AIFA). Briefly, in December 2021 (https://www.aifa.gov.it/emergenza-covid-19, accessed on 18 January 2025) [15], to be eligible for the study, participants had to have a positive nasopharyngeal swab (NPS) test for SARS-CoV-2 infection by antigenic or molecular methods, mild or moderate COVID-19 symptoms for no more than 7 days, and at least one of the following risk factors for worsening disease: a body mass index (BMI) of 35 or higher, chronic peritoneal dialysis or hemodialysis, diabetes mellitus that was uncontrolled or complicated, oncologic/onco-hematologic pathology in the active phase, and primary or secondary immunodeficiency. Patients who were aged 55 years or older were also qualified in the case of any cardio-cerebrovascular diseases, chronic obstructive pulmonary disease (COPD), or other chronic respiratory diseases. Patients requiring hospitalization for COVID-19 or supplemental oxygen therapy were excluded.

In June 2022, the AIFA expanded the use of early therapy for COVID-19 outpatients, including all patients with one of the following: age 65 or older; a BMI above 30; any chronic kidney impairment (including those on peritoneal dialysis or hemodialysis); uncontrolled or complex diabetes mellitus; any condition that weakens the immune system, including primary and secondary immunodeficiencies; heart or brain blood vessel diseases (including high blood pressure with organ damage); COPD or other chronic lung diseases; chronic liver disease; blood disorders; oncologic/onco-hematologic pathology in the active phase; nerve development; and degeneration disease.

The Monoclonal Antibodies Screening Score [MASS (7–22)] was used to measure comorbidity burden. This score gives points based on the following factors: age ≥ 65 (2 points), BMI ≥ 35 (1 point), diabetes mellitus (2 points), chronic kidney disease (3 points), cardiovascular disease in a patient ≥ 55 years (2 points), chronic respiratory disease in a patient ≥ 55 years (2 points), hypertension in a patient ≥ 55 years (1 point), and immunocompromised status (3 points).

### 2.1. Early Anti-SARS-CoV-2 Therapies

Bamlanivimab/Etesevimab (700 mg/1400 mg), Casirivimab/Imdevimab (1200 mg/1200 mg), or Sotrovimab (500 mg) was administered by one-hour intravenous infusion, and patients were observed for one hour after infusion. Remdesevir at 200 mg was administered iv on day 1 and at 100 mg from days 2 to 3. Additionally, 300 mg of Nirmatrelvir, with 100 mg of Ritonavir, was administered daily for a total of 5 days. The treatment decision was based on the availability of therapies and clinical judgment.

The primary endpoint was the time to negative NPS for SARS-CoV-2 from symptom onset. The secondary endpoints included (a) the time to COVID-19-related hospitalization or death by day 30 and (b) the time to all-cause hospitalization or death by day 30.

### 2.2. Data Collection

At INMI, COVID-19 outpatients had scheduled visits on days 1 (D1), 7 (D7), and 30 (D30). At Santa Maria Goretti University Hospital, outpatients had a scheduled visit on D1 only and then a phone follow-up on D7 and D30. At each visit, medical evaluation, vital signs measurement, laboratory tests, and adverse effects reporting were performed. If patients did not attend personal visits, they were contacted by phone to check their clinical conditions. As this was a real-life study, different methods were used to measure serology and virological parameters depending on the laboratory workload and availability of assays during the COVID-19 pandemic.

The collected data included age, sex, first positive NPS, symptom onset, peripheral oxygen saturation (SpO2), body mass index (BMI), comorbidities (such as hematological disorders, oncological disorders, diabetes, obesity, hypertension, chronic lung disorders, cardiovascular disorder, cerebrovascular disorders, and kidney failure and/or autoimmune disorders), the time from the last administration of any immunosuppressive drugs (TID), the number of COVID-19 vaccine doses received, the time to treatment (TTT), the interval between symptom onset and the administration of COVID-19 therapy, the time to negative test (TNT), the time to negative NPS for SARS-CoV-2 from the first positive NPS, and clinical outcomes (hospitalization and death) for each participant.

A real-time reverse transcription polymerase chain reaction (RT-PCR) test was evaluated at enrollment before starting early anti-COVID-19 therapy. It was performed according to the laboratory workflow using different platforms: the DiaSorin Simplexa^®^ COVID-19 Direct platform, the Abbott m2000 RealTime System, and the Cobas^®^ SARS-CoV-2 Test on the fully automated Cobas^®^ 6800 System. Viral characterization was performed on the NPS samples collected, when possible, by Next-Generation Sequencing (NGS) on the Ion Torrent Platform using the Ion AmpliSeq SARS-CoV-2 Research Panel, as per the manufacturer’s instructions.

### 2.3. Statistical Analysis

The Shapiro–Wilk test was employed to assess data normality. A power analysis was performed by comparing two independent proportions and the median of two unmatched groups, imposing an α error probability of 0.05 and a power of (1-β) of 0.95. Moreover, Cohen’s d value was calculated to estimate the effect size. The power analysis and the effect size were estimated using GPower 3.1 statistical software [16].

The Kolmogorov–Smirnov test was used to examine possible statistically significant differences among age, SpO2, BMI, TTT, TID, and TNT, while the chi-squared test was used to explore possible statistically significant differences between the prevalence of comorbidities, the number of COVID-19 vaccine doses received, and clinical outcomes.

An odds ratio calculation was performed to find an eventual correlation between comorbidities and mortality, treatment with MoAbs or AVAs, and hospitalization and death during the 30-day follow-up.

The time that SARS-CoV-2 NPS was detectable in days was compared among patients who had not been vaccinated, received I–II doses, or received III–IV doses using the Kruskal–Wallis test and the Dunn post hoc test. Additionally, the same tests were used to compare the length of time that SARS-CoV-2 NPS was detectable in days among patients who were infected with alpha, delta, or omicron VoCs based on the date of infection and the epidemiological prevalence of these VoCs in Italy during the study period.

To control for possible confounding factors, a logistic regression model was applied using death, hospitalization, and time of positivity as outcomes and the type of therapy, time from the last immunosuppressive therapy, number of vaccinations, and MASS as potential variables. To compare the time from vaccination, TID, TNT, TTT, hospital admission, positive NPS after D7, positive NPS after D30, and death, a Receiving Operational Curve (ROC) analysis was used, and cut-off values were identified according to the corresponding AUC. Statistical analyses were conducted using MedCalc^®^ Statistical Software version 20 (MedCalc Software Ltd., Ostend, Belgium; https://www.medcalc.org; 2021) and GraphPad Prism version 9.0.0 for Windows (GraphPad Software, San Diego, CA, USA, www.graphpad.com).

## 3. Results

### 3.1. Descriptive Statistics

A total of 1472 patients were enrolled from 31 March 2021 to 31 March 2022. The median age of the study population was 58 years; of them, 665 (45%) were male, 149 (10.7%) were unvaccinated, and 886 (60.1%) were fully vaccinated (Table 1). The COVID-19 patients were defined as immunocompromised in the presence of primary or secondary immunodeficiency (517 patients, 35%), oncological disorders (561 patients, 38.1%), and hematological disorders (394 patients, 16.7%). An analysis of additional comorbidities revealed a higher prevalence of cardiovascular diseases (402, 27.3%), followed by cerebrovascular diseases (342, 23.2%) and chronic lung diseases (188, 12.8%) (Table 1).

A total of 688 patients (46%) received MoAb treatment, while the remaining 783 (54%) received AVAs. In the AVA group, a significantly higher prevalence (*p* < 0.01) of combined IC disorders due to underlying autoimmune, oncological, and/or hematological disorders was reported than in the MoAb group.

The patients receiving AVAs were significantly older and had a higher prevalence of cardio-cerebral disorders and obesity than the MoAb treatment group (Table 1). Conversely, the patients treated with MoAbs displayed a statistically greater prevalence of hypertension and renal dysfunction. The MoAb group received treatment a median of one day earlier than the AVA group (*p* < 0.001). Interestingly, the MASS, a measure of disease severity, was similar between both groups, with a value of 3 in each.

The MoAb group had a significantly higher proportion of unvaccinated or partially vaccinated patients than the AVA group (*p* < 0.05). The body mass index (BMI) was significantly higher in the AVA group than in the MoAb group, with a large effect size (25.4 vs. 24.3 days, *p* < 0.05, Cohen’s d = 1). The TTT was significantly shorter in the AVA group than in the MoAb group, with a large effect size (3 vs. 4 days, *p* < 0.05, Cohen’s d = 5.65). The TID was significantly shorter in the AVA group than in the MoAb group, with a very small effect size (7.6 vs. 7.2 days, *p* < 0.05, Cohen’s d = 0.01). The TNT was significantly lower in the AVA group than in the MoAb group, with a medium effect size (11 vs. 17 days, *p* < 0.05, Cohen’s d = 0.49). No statistically significant differences were observed in hospital admission or COVID-19-related deaths between the treatment groups.

### 3.2. Potential Risk Factors Associated with Positive SARS-CoV-2 NPS on D7 and D30, COVID-19 Hospitalization, and Death

The odds ratio (OR) analysis revealed that the patients treated with MoAbs had a significantly higher likelihood of testing positive for SARS-CoV-2 by NPS on day 7 (OR: 3, 95% CI: 1.72–5.23, *p* < 0.01) and day 30 (OR: 6, 95% CI: 3.7–10.5, *p* < 0.01) than those receiving AVAs.

No statistically significant differences were observed in hospital admission rates between the two patient groups (Table 2). However, a non-significant increasing trend in hospitalization risk in the MoAbs group was reported. Due to the low number of deaths recorded in the study (a total of five patients), an odds ratio analysis of mortality was not possible.

Regarding the OR analysis, hypertension emerged as a significant risk factor for mortality within the MoAb group (OR: 8.84, 95% CI: 1.42–55.0, *p* < 0.05). In contrast, no statistically significant correlations were found between the number of vaccine doses and clinical outcomes such as positive NPS on day 30, hospitalization, or death (all *p* > 0.05).

### 3.3. Last Administration of Immunosuppressive Drug as Potential Predictor of Positive SARS-CoV-2 NPS on Days 7 and 30

**Figure 1:**
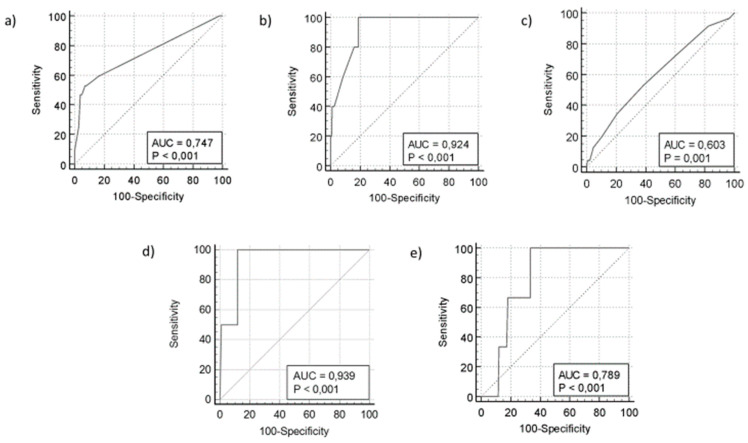
ROC curve to calculate the (**a**) accuracy of days from the last immunosuppressive drug as a predictor of prognostic factors of positive NPS, (**b**) risk of hospitalization based on time from the last immunosuppressive drug, (**c**) predictive value of the time to antiviral agent (AVA) or monoclonal antibody (MoAb) treatment initiation, (**d**) predictive value of treatment initiation within 72 h and hospital admission compared to TID, (**e**) predictive value of last vaccination in terms of mortality.

The ROC analysis revealed that patients who received immunosuppressive drugs within 13 days prior to SARS-CoV-2 infection had a higher probability of a positive NPS on day 30 (AUC = 0.747, 95% CI: 0.663–0.819, sensitivity = 53%; specificity = 93%, *p* < 0.001) (Figure 1a). Similarly, those who received immunosuppressive drugs within 17 days of infection had an increased risk of hospitalization (AUC = 0.924, 95% CI: 0.894–0.948, sensitivity = 100%, specificity = 81.2%, *p* < 0.0001) (Figure 1b).

Treatment initiation timing (TTT) also influenced outcomes; patients who started treatment more than 72 h after symptom onset were more likely to test positive on day 30 (AUC = 0.603, 95% CI: 0.570–0.635, sensitivity = 53%, specificity = 61%, *p* < 0.001) (Figure 1c). Further analysis confirmed that immunosuppressive drugs administered within 14 days of infection strongly predicted positive NPS on day 30 (AUC = 0.939, 95% CI: 0.874–0.976, sensitivity = 100%; specificity = 88.24%, *p* < 0.0001) (Figure 1d).

A shorter time between vaccination and SARS-CoV-2 infection was associated with better clinical outcomes, including a lower mortality risk (AUC = 0.789, 95% CI: 0.742–0.831, sensitivity = 100%; specificity = 67%, *p* < 0.001) (Figure 1e). Fully vaccinated patients (three or more doses) had significantly shorter durations of positive NPSs than unvaccinated or partially vaccinated individuals (median duration: 11 days vs. 19 days and 17 days, *p* = 0.0001 and *p* < 0.0001, respectively). Among the unvaccinated patients, those treated with AVAs had shorter NPS durations than those treated with MoAbs (23.98 vs. 13.52 days, *p* < 0.01).

**Figure 2:**
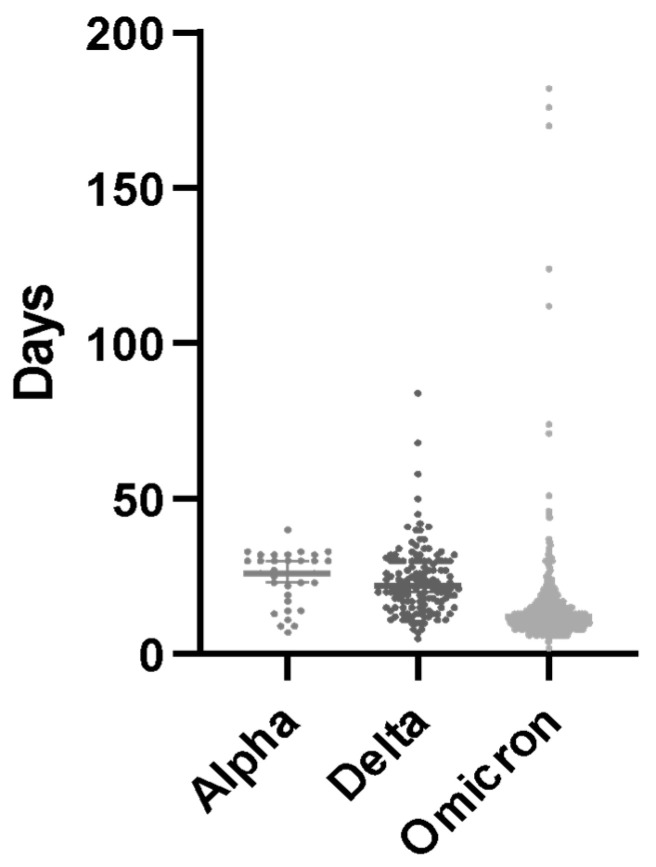
Positive nasopharyngeal swab persistence of the different VoCs.

The duration of positive NPSs differed among patients infected with alpha, delta, and omicron variants (median: 25 vs. 24 vs. 14 days, *p* < 0.01). A post hoc analysis revealed significant differences between alpha and omicron (*p* < 0.01) and delta and omicron (*p* < 0.01) (Figure 2).

Logistic regression identified treatment timing and type as key predictors of hospitalization (*p* < 0.01, Chi-squared = 14.64, R^2^ = 0.47, AUC = 0.98, 95% CI: 0.95–0.99). MoAb treatment was associated with a significantly lower odds ratio for hospitalization (OR: 0.0001, 95% CI: 0.0–0.1), while delayed treatment (TTD) increased hospitalization risk (OR: 1.61, 95% CI: 1.18–2.2). Vaccination status became statistically significant after TTD was omitted. However, this apparent “boost” in significance resulted from the fact that the vaccination variable exhibited virtually no within-group variance, with only three overall events, which severely compromised coefficient estimate stability and encouraged overfitting. Hence, we were careful to retain TTI in our primary multivariable model for both its powerful mechanistic relevance to viral clearance and to avert artefactual inflation of the vaccination effect under sparse data conditions.

## 4. Discussion

This analysis is the first real-world evaluation of two well-established anti-SARS-CoV-2 agents, antivirals and monoclonal antibodies, in IC outpatients at two Italian COVID-19 reference hospitals. The study was conducted when the different VoCs detected during the study period were still fully susceptible to MoAb therapy.

Immune-compromised COVID-19 patients have shown persistent SARS-CoV-2 infection, high rates of hospitalization for severe disease, and increased case fatality ratios [17]. Despite clear evidence of the increased vulnerability of IC patients exposed to SARS-CoV-2 infection, there is no specific targeted therapeutic approach for this population [10].

This study shows that AVA treatment reduced the chance of a positive SARS-CoV-2 NPS on day 30 in IC patients with SARS-CoV-2 infection, compared to MoAb treatment. No significant differences were observed in hospitalization or death rates between the groups.

Patients receiving AVAs were older and had more underlying comorbidities, and a higher percentage of them had previous SARS-CoV-2 vaccinations. However, they had a similar MASS to those undergoing MoAb treatment. Interestingly, the AVA-treated patients received COVID-19 therapy earlier and had a three and six times lower chance of a positive NPS on D7 and D30, respectively, than the MoAb-treated patients. However, the groups did not differ significantly in hospitalization or death rates.

The best time to treat SARS-CoV-2 is as soon as symptoms appear, since this matches the disease’s pathophysiology. Indeed, the first days of symptoms mark the viral phase, where the virus invades and multiplies [18]. AVAs inhibit viral replication, and the optimal approach is to administer AVAs early in the infection process before the virus reaches its highest level of replication [19]. In our group, treatment within 72 h of symptom onset reduced the chance of a positive NPS on day 30 for both AVAs and MoAbs.

The timing of the last administration of the immune-suppressive drug appears to influence the duration of a positive SARS-CoV-2 NPS. Patients who had their last immunosuppressive dose more than 13 days before the first positive NPS had a higher probability of having a positive NPS on D30 and of being hospitalized. This supports the idea that immunosuppression slows down viral clearance, possibly causing longer viral shedding. Also, starting treatment after 72 h from the first positive NPS and within 14 days from the last immunosuppressive drug dose seems to be linked to a higher risk of hospital admission. This finding highlights the importance of early and timely intervention to reduce severe or long-lasting illness in IC patients.

Being fully vaccinated reduces the chances of testing positive for NPS after 30 days [20,21]. However, in our study, vaccination did not have a significant effect on 30-day NPS positivity, even though there were more vaccinated patients in the AVA group. Studies have shown that COVID-19 vaccination in people with compromised immunity provides substantial protection against hospitalization, ICU admission, and hospital death [3]. Consistent with these results, our data report a potential link between a shorter time interval between vaccination and SARS-CoV-2 infection and a lower mortality risk.

The probability of a positive NPS on D30 seems to also be affected by the VoC. Our results indicate that the omicron VoC may have a lower chance of prolonged positivity to D30 in IC individuals than the alpha VoC, in accordance with previous data [22,23]. Based on the results of our analyses, the type of VoC circulating at the time of infection is certainly a possible confounding factor; however, given the large number of patients enrolled, it is likely that this had a relative impact on the results of the analyses. Indeed, the aim is precisely to establish which causes and risk factors may predispose to a prolonged duration of infection and thus be less affected by the median duration due to VoC.

This study is limited by several factors. The lack of information regarding specific immunosuppressive and underlying patient disorders does not allow for the identification of specific patient or disease clinical features but rather only general trends within IC patients infected by SARS-CoV-2. Additionally, reliance on antigenic testing on D30 may have underestimated positive results, but this risk is well balanced between groups. Another limitation is that hospitalization and death have only a few overall events, which severely compromise coefficient estimate stability and encourage overfitting. Hence, we performed several statistical analyses in order to avert artefactual inflation of the vaccination effect under sparse data conditions.

Seasonal patterns of community-circulating SARS-CoV-2 variants were used as an epidemiologic reference for the VoC of each single patient, and, finally, potential variability in hospital admission criteria across centers could not be excluded. Despite these limitations, this report highlights the protective role of recent SARS-CoV-2 vaccination and early antiviral treatment in IC individuals.

Based on our findings, immunocompromised patients should receive COVID-19 vaccination and promptly undergo early antiviral therapy to prevent clinical progression and the persistence of SARS-CoV-2 infection.

## 5. Conclusions

The prompt initiation of AVA therapy in IC patients was associated with a significantly lower probability of sustained detection at later intervals (7 and 30 days) when compared with MoAbs, even though AVA recipients were older and suffered from greater comorbidity burdens. The initiation of treatment within 72 h of symptom onset markedly improved virologic outcomes with both AVA and monoclonal antibodies, indicating that the concordance of therapeutic timing with the viral replication phase is critical. Also, shorter intervals between recent vaccination and infection and the recent immunizations suggested that there was less likelihood of prolonged viral shedding, thus adding more value to up-to-date vaccination in this vulnerable population.

Indeed, the interval that had elapsed since the last dose of immunosuppression proved to be significant in the prediction of both the prolonged isolation of SARS-CoV-2 and the risk of admission to hospital. The observed variation in viral clearance by specific strain—most notably a shorter period of positivity with omicron than with alpha—did not mask the overriding impact of early antiviral intervention and vaccination status. Limitations such as dependence on antigen testing at 30 days, indirect allocation by variant ID, and potential variability in admissions criteria from center to center indicate that prospective studies with complete immunologic and virologic profiling are warranted.

Our findings provide a case for a strategy to manage immunocompromised patients, which is designed around the timely initiation of antiviral treatment—in ideal cases, within 72 h of symptom presentation—and complete, up-to-date vaccination against SARS-CoV-2. Such an approach would offer the best chance of preventing both prolonged viral replication and progression to severe COVID-19 among this very high-risk group and could possibly be a good model for investigating the role of antiviral therapy during viral infection in IC patients.

## Figures and Tables

**Table 1:** Characteristics of study population.

	Whole (n° 1472)	MoAbs (n° 688)	AVAs (n° 783)	*p*-Value
Age, median (IQR)	58 (48–69)	57 (46–67)	61 (51–72)	*p* < 0.01
Variant of concern, no. (%)	alpha, 76 (5%) delta, 283 (19%) omicron, 1113 (75%)	alpha, 70 (10%) delta, 282 (41%) omicron, 336 (49%)	alpha, 6 (1%) delta, 1 (0.1%) omicron, 776 (99%)	
Treatment, no. (%)	1472	688 (46%)	783 (54%)	
		Bamlanivimab 161 (23%)	Remdesevir 249 (31%)	
		Bamlanivimab/Etesevimab 65 (9%)	Nirmatrelvir/r 271 (34%)	
		Casirivimab/imdevimab 131 (19%)	Molnupiravir 263 (33%)	
		Sotrovimab 331 (48%)		
Male (% whole population)	665 (45%)	301 (44%)	364 (47%)	n.s.
BMI (IQR)	24.83 (22.49–27.75)	24.30 (22.36–27.41)	25.39 (22.54–27.82)	<0.01
TTI, median days (IQR)	3 (2–4)	4 (2–5)	3 (2–4)	<0.001
TID median days (IQR)	7 (7–9)	7 (7–29)	7 (7–7)	<0.001
Cause of IC status				
Hematological disorders, no. (%)	394 (26.7%)	161 (23.4%)	233 (29.7%)	<0.01
Oncological disorders, no. (%)	561 (38.1%)	223 (32.4%)	338 (43.2%)	<0.01
Primary or secondary immunodeficiency, no. (%)	517 (35%)	304 (44.1%)	212 (27%)	<0.01
		Comorbidities		
Diabetes, no. (%)	124 (8.4%)	56 (8.1%)	68 (8.6%)	n.s.
Obesity, no. (%)	158 (10.7%)	65 (9.4%)	93 (11.8%)	<0.01
Hypertension, no. (%)	154 (10.5%)	90 (13%)	64 (8.1%)	<0.01
Chronic lung disorders, no. (%)	188 (12.8%)	80 (11.6%)	108 (13.7%)	n.s.
Cardiovascular disorders, no. (%)	402 (27.3%)	144 (20.9%)	258 (32.9%)	<0.01
Cerebrovascular disorders, no. (%)	342 (23.2%)	113 (16.4%)	229 (29.2%)	<0.01
Renal failure, no. (%)	103 (7%)	65 (9.4%)	38 (4.8%)	<0.01
MASS, median	3 (3-3)	3 (3-4)	3 (3-3)	n.s.
COVID-19 vaccination				
None, no. (%)	149 (10.1%)	105 (15.2%)	43 (5.4%)	<0.05
I dose, no. (%)	39 (2.6%)	29 (4.2%)	10 (1.2%)	<0.05
II doses, no. (%)	298 (22.2%)	236 (34%)	61 (7.7%)	<0.05
III doses or more, no. (%)	886 (60.1%)	266 (38.6%)	599 (76.5%)	<0.05
Clinical outcome				
TNT, median days (IQR)	13 (10–19)	17 (12–26)	11 (9–14)	<0.05
Admission to hospital, no. (%)	31 (2.1%)	20 (2.9%)	11 (1.6%)	<0.05
Death, n° (%)	5 (0.34%)	4 (0.58%)	1 (0.15%) (1)	<0.05

**Table 2:** Odds ratio analysis between patients who received AVAs and MoAbs.

Clinical Features	MoAbs	AVAs
Positive NPS on D7 OR (95% CI), *p*-value	3 (1.72–5.23), *p* <0.01	0.33 (0.19–0.58), *p* < 0.01
Positive NPS on D30 OR (95% CI), *p*-value	6 (3.7–10.5), *p* < 0.01	0.16 (0.09–0.27), *p* < 0.01
Hospital admission OR (95% CI), *p*-value	1.78 (0.98–3.23), *p* = 0.07	0.56 (0.3–1.02), *p* = 0.07
All-cause death for all ORs (95% CI), *p*-value	1.1 (0.41–2.95), *p* > 0.05	0.96 (0.34–2.42), *p* > 0.05

## Data Availability

Anonymized participant data will be made available upon reasonable request directed to the corresponding author.
